# Risk factors associated with depression among French university students during covid-19 lockdown: Thibaut Sabatier

**DOI:** 10.1093/eurpub/ckac130.188

**Published:** 2022-10-25

**Authors:** T Sabatier, C Godefroy, MP Tavolacci, J Ladner

**Affiliations:** INSERM UMR 1073, Rouen University, Rouen, France

## Abstract

**Background:**

During the COVID-19 pandemic students’ lives changed drastically, especially regarding their mental health. Social isolation, induced by lockdown, could be the cause of the development of mental disorders in this population. Therefore, the aim of this study was to identify factors associated with depression during French first Covid-19 lockdown among university students.

**Methods:**

This cross-sectional study, which is a part of the COVID-19 International Student Well-Being Study (ISWS consortium), used the validated CES-D 8 score (Center for Epidemiologic Studies - Depression Scale) to measure depression levels. Data on socio-demographics, curriculum, living condition, academic environment and social interactions were collected few days after the first lockdown in France, from 13 to 31 May 2020. The potential impact of risk factors on depression was studied by multinomial logistic regression.

**Results:**

A total of 3593 students were included. The CES-D 8 mean score was 8.65 (SD = 5.08). Literature students had the highest average CES-D 8 score (9.47, SD = 5.16). Independent factors associated with the higher scores of depressions included having limited financial resources (AOR=2.49, 95% CI = 1.84-3.38) having academic concerns, including students worried about not completing the academic year (AOR=2.93, AOR=2.37-3.64) and stressed with changes in teaching methods (AOR=3.55, 95% CI = 2.82-4.46). Otherwise, living with parents during lockdown and being in a relationship were significant protective factors against depression.

**Conclusions:**

This study highlights the impact of changing social network, living conditions, and academic environment on depression among university students. Preserving students from social isolation must be a critical priority for universities. Future universities’ policy strategy could combine on-site teaching with online courses and consider the role of students’ social contacts, with a particular emphasis on mental health.

**Key messages:**

- Social isolation is an important risk factor of depression in students.
- Implications for policy makers demonstrating the need for effective mental health programs and guidance as a public health strategy in universities.

